# The roles of HIV-1 specific CD8+ T cell responses and HLA class I alleles on viral control and viral escape in HIV-1 infected Thai individuals

**DOI:** 10.1186/1742-4690-9-S2-P248

**Published:** 2012-09-13

**Authors:** S Buranapraditkun, U Hempel, WH Hildebrand, TM Allen, K Ruxrungtham

**Affiliations:** 1 Chulalongkorn University, Bangkok, Thailand; 2 Ragon Institute of MGH, MIT and Harvard, Boston, MA, USA; 3 Department of Microbiology and Immunology, Univ. of Oklahoma, Oklahoma City, OK, USA

## Background

Knowledge about the role of specific HLA class I alleles, CD8+ T cell responses, and viral escape on viral control have not been well characterized in clade CRF01_AE and Asian ethnics.

## Methods

195 naïve HIV-1 CRF01_AE infected Thai individuals were screened for HIV-1 specific CD8+ T cell responses with a set of 413 OLPs of the HIV-1 proteome by IFN-gamma ELISpot assay. Novel epitopes were characterized by HLA restriction and fine epitope mapping. The association of epitopes and/or HLA alleles with low VL level and/or viral escape was analyzed.

## Results

Thirty-three OLPs were identified as potential novel epitopes. A viral control epitope, RI10 (HIV-protease, previously described in HIV-1 B clade as -B*13 restricted) was found restricted by HLA-A*0203 in Thais. Interestingly, HLA-A*0203+ve patients with RI10 responders had a significantly lower VL than non-responders (p = 0.0167). This data may support the low VL from loss of viral fitness of RI10. Moreover, the patients exhibiting mutations in RI10 showed no ELISpot responses. Another known HLA-A*1101 epitope, AK11 (in Gag-p24) was also identified as a viral control epitope in this study. Of note, there was no significant mutation found in patients expressing A*1101. We also found a novel immunodominant epitope (29% response rate) restricted by HLA-Cw*0102: YI9 (in Gag-p24) which was associated with viral escape. Mutations at P2 (S278X), P4 (V280X) and P5 (S281G) impaired the Elispot responses, however the P2 anchor S278K mutation had the highest negative impact (p = 0.0002).

## Conclusion

In HIV-1 CRF01_AE infected Thais we characterized three CD8 epitopes (RI10, AK11 and YI9) restricted by HLA-A*0203, -A*1101 and -Cw*0102, respectively. RI10 and AK11, but not YI9, were associated with lower VL and possible control of HIV. Further characterization of those possible novel epitopes is warranted.

